# Optimizing hexanoic acid biosynthesis in *Saccharomyces cerevisiae* for the de novo production of olivetolic acid

**DOI:** 10.1186/s13068-024-02586-2

**Published:** 2024-12-04

**Authors:** Kilan J. Schäfer, Marco Aras, Eckhard Boles, Oliver Kayser

**Affiliations:** 1 Faculty of Biochemical and Chemical Engineering, Technical University Dortmund, 44227 Dortmund, Germany; 2 Institute of Molecular Biosciences, Goethe-University Frankfurt, 60438 Frankfurt am Main, Germany

**Keywords:** Hexanoic acid, Hexanoyl-CoA, Medium-chain fatty acids, Fatty acid synthase, Reverse beta oxidation pathway, Olivetolic acid, Cannabinoids, Pantothenate kinase

## Abstract

Medium chain fatty acids (MCFAs) are valuable platform compounds for the production of biotechnologically relevant chemicals such as biofuels and biochemicals. Two distinct pathways have been implemented in the yeast *Saccharomyces cerevisiae* for the biosynthetic production of MCFAs: (i) the mutant fatty acid biosynthesis (FAB) pathway in which the fatty acid synthase (FAS) complex is mutated and (ii) a heterologous multispecies-derived reverse β-oxidation (rBOX) pathway. Hexanoic acid has become of great interest as its acyl-CoA ester, hexanoyl-CoA, is required for the biosynthesis of olivetolic acid (OA), a cannabinoid precursor. Due to insufficient endogenous synthesis of hexanoyl-CoA, recombinant microbial systems to date require exogenous supplementation of cultures with hexanoate along with the overexpression of an acyl-CoA ligase to allow cannabinoid biosynthesis. Here, we engineer a recombinant *S. cerevisiae* strain which was metabolically optimized for the production of hexanoic acid via the FAB and rBOX pathways and we combine both pathways in a single strain to achieve titers of up to 120 mg L^−1^. Moreover, we demonstrate the biosynthesis of up to 15 mg L^−1^ OA from glucose using hexanoyl-CoA derived from the rBOX pathway.

**Supplementary Information:**

The online version contains supplementary material available at 10.1186/s13068-024-02586-2.

## Introduction

Medium chain fatty acids (MCFAs) are carboxylic acids which contain a saturated carbon chain of length C_6_ to C_12_. They are industrially valuable compounds with a broad range of applications as they have direct uses as constituents of antimicrobial agents [1, 2] and can serve as platform chemicals for the production of biofuels, biochemicals or pharmaceuticals, for example in drug delivery systems [3–5]. Various microbial systems have been adopted or engineered to allow the sustainable and cost-efficient production of MCFAs by fermentation from biomass as an alternative to the use of unsustainable and environmentally harmful sources for MCFA extraction such as fossil resources and plant oils. Natural MCFA producers include the bacterial species *Clostridium kluyveri* and *Megasphaera elsdenii* which use the reverse β-oxidation (rBOX) pathway to anaerobically elongate short-chain organic acids to MCFAs and both have been exploited in biotechnological processes [6–10]. Other microorganisms have been engineered as synthetic MCFA producers and include the prominent microbial chassis organisms *E. coli* and the yeast *Saccharomyces cerevisiae*. Several strategies have been implemented in these organisms to synthesize MCFAs, however, two main metabolic pathways are most commonly used: the endogenous fatty acid biosynthesis (FAB) pathway or the heterologous reverse β-oxidation (rBOX) pathway [11–17].

In *S. cerevisiae*, engineering of the endogenous FAB pathway for MCFA production can be achieved by modifying the type I fatty acid synthase (FAS), a large 2.6 MDa multidomain enzyme complex which is responsible for the biosynthesis of long chain fatty acids (LCFAs) [18, 19]. FAS is encoded by two genes, *FAS1* and *FAS2*, which encode the β- and α-subunits, respectively. Six β-subunits and six α-subunits assemble into a heterododecameric complex which contains distinct catalytic domains responsible for the elongation and reduction of growing acyl-CoA chains using acetyl-CoA as the starting unit and malonyl-CoA as the elongation unit [20]. The growing acyl chain is covalently linked to a flexible acyl carrier protein (ACP) domain which shuttles the substrate between the active centers of the enzyme complex in order to elongate the chain and fully reduce the β-keto group through a series of sequential reduction steps [21]. Each cycle consumes two molecules of NADPH and the cycle is repeated until the acyl chain reaches a length of C_16_ or C_18_, after which it is released as an acyl-CoA [18]. Rational enzyme engineering approaches have led to the identification of specific residues involved in chain length control which can be mutated in order to shift the product spectrum of fatty acids towards medium-chain fatty acyl-CoAs which are subsequently cleaved via endogenous thioesterase (TE) activity and secreted as free MCFAs [16].

Alternatively, MCFAs and their derivatives can be synthesized using a multispecies-derived rBOX pathway [22, 23]. This pathway, first described using *E. coli* as a heterologous host, utilizes two molecules of acetyl-CoA which are condensed to form acetoacetyl-CoA via the action of a thiolase or β-ketoacyl-CoA synthase. Next, the β-keto group is subject to a series of reduction steps, similar to the FAB pathway. A β-ketoacyl-CoA reductase reduces the β-ketoacyl-CoA group to a hydroxyl group, using one molecule of NAD(P)H which is in turn dehydrated to form an enoyl-CoA by a β-hydroxyacyl-CoA dehydratase. Finally, an enoyl-CoA reductase reduces the enoyl-CoA to form butyryl-CoA (C_4_), using a second molecule of NAD(P)H. The rBOX pathway has since been implemented in *S. cerevisiae* for the production of a range of compounds and optimized for n-butanol production [12, 24]. Moreover, the cycle can be extended to form hexanoyl-CoA (C_6_) by incorporating a β-ketothiolase (*bktB*) from *Cupriavidus necator* which is able to accept both butyryl-CoA and acetyl-CoA as substrates [11] and has recently been further extended to octanoyl-CoA (C_8_) in *S. cerevisiae* by using an alternative β-hydroxyacyl-CoA dehydratase, thereby showing that bktB has the capacity to accept hexanoyl-CoA as a substrate [25]. The medium-chain fatty acyl-CoAs are finally hydrolyzed by endogenous TEs, similar to during FAB-mediated production.

Most studies on microbial MCFA production to date have focused on the synthesis of octanoic or decanoic acids and their derivatives or describe the production of different chain length mixtures. In contrast, little work has focused on the selective production of hexanoic acid, especially in *S. cerevisiae*. Nevertheless, the selective production of hexanoic acid has become of great interest in recent years as its acyl-CoA ester, hexanoyl-CoA, is a key precursor for the biosynthetic production of cannabinoids. Some studies have described the selective biosynthesis of hexanoic acid in *E. coli*, reaching titers of up to 528 mg L^−1^, or the thermotolerant yeast *Kluyveromyces marxianus*, reaching titers of up to 154 mg L^−1^ [26, 27]. Recently, the selective production of up to 75 mg L^−1^ hexanoic acid was described in a metabolically optimized *S. cerevisiae* strain via the rBOX pathway [25].

Cannabinoids are a class of prenylated polyketides, found naturally in the plant species *Cannabis sativa*, the most common of which are ∆^9^-tetrahydrocannabinol (∆^9^-THC) and cannabidiol (CBD). Using microbial systems for cannabinoid production has attracted a wealth of research and commercial attention in recent years due to an increasing global demand and the advantages over extraction from the plants. Cannabinoid synthesis starts from hexanoyl-CoA and geranyl pyrophosphate (GPP). In 2019, Luo and coworkers were able to reconstitute the complete cannabinoid biosynthesis pathway in *S. cerevisiae* for the first time by overexpressing a heterologous rBOX pathway for the synthesis of hexanoyl-CoA and overexpressing and modifying the mevalonate pathway for the production of GPP [28]. Following this, overexpression of the *C. sativa*-derived genes encoding a type III polyketide synthase, olivetol synthase (OLS), sometimes referred to as tetraketide synthase (TKS) and olivetolic acid cyclase (OAC) allowed the production of olivetolic acid (OA). OA is subsequently prenylated with GPP by an aromatic prenyltransferase (PT) to form cannabigerolic acid (CBGA), the central precursor for the production of various cannabinoids [29]. CBGA is finally converted to either ∆^9^-tetrahydrocannabinolic acid (THCA) or cannabidiolic acid (CBDA), depending on the expression of THCA synthase or CBDA synthase [28]. Following exposure to heat, THCA and CBDA are decarboxylated in a non-enzymatic reaction to form the final cannabinoids, THC or CBD, which exert pharmacological effects [30]. Recent publications describe the improvement of this pathway to overcome various metabolic limitations and increase the production titers of CBGA [31, 32]. Despite overexpression of the rBOX pathway in these studies, the endogenous production of hexanoyl-CoA remained low and higher titers of cannabinoids or their precursors were only achieved upon supplementing the cultures with between 0.5 and 3 mM hexanoic acid and overexpressing a *C. sativa*-derived acyl-CoA ligase (*AAE1*) [28, 31–33]. However, feeding of hexanoic acid is unfavorable in industrial settings as this would complicate process design and increase costs. Furthermore, in consideration of the emergence of future cannabinoid producing strains able to turnover greater concentrations of hexanoic acid, toxicity is likely to become a major limitation, as is the case with other MCFAs [34].

**Fig. 1:**
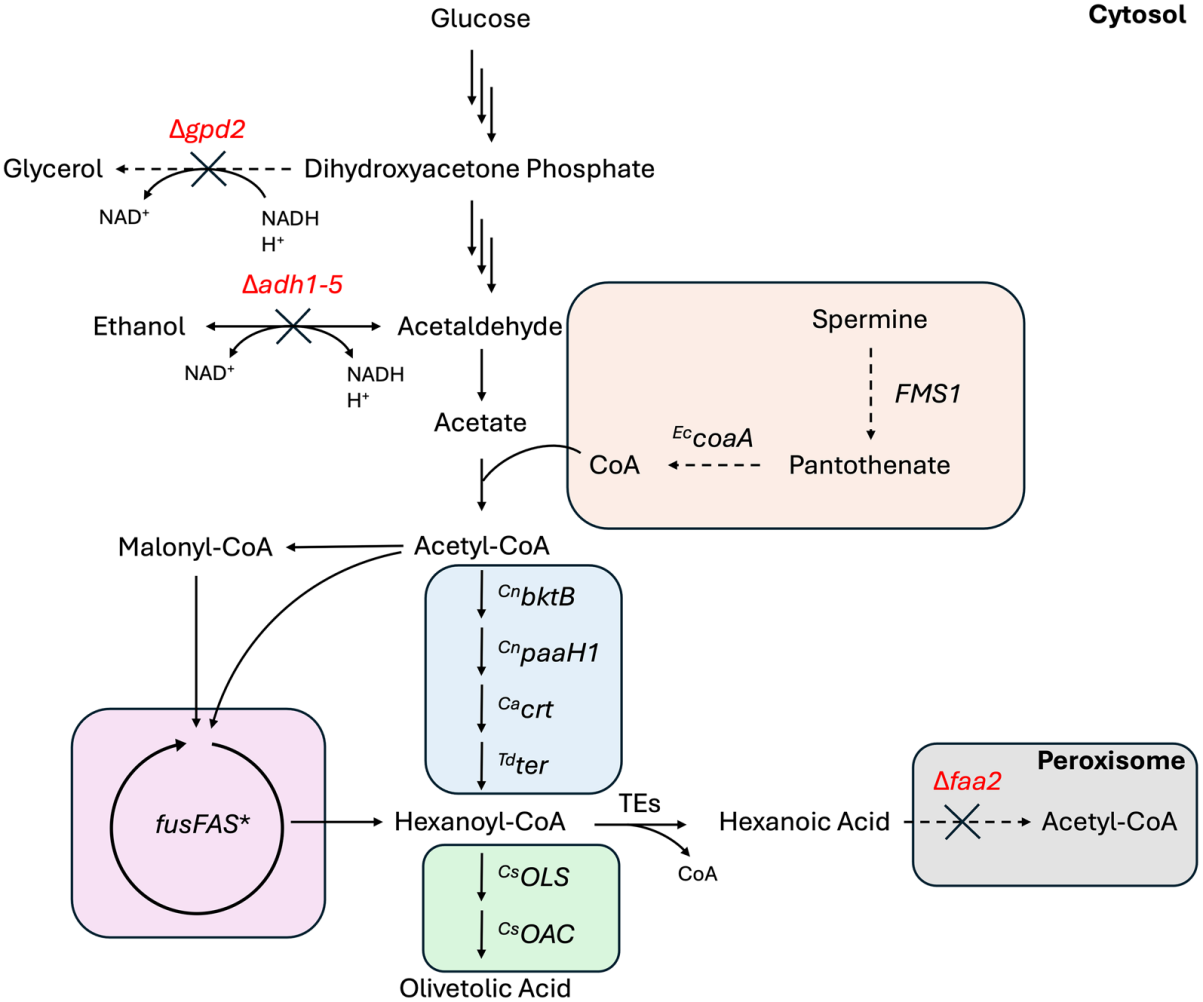
Schematic of engineered metabolic pathways for hexanoic acid and olivetolic acid biosynthesis in *S. cerevisiae*. CoA biosynthesis pathway (orange), rBOX pathway (blue), FAB pathway (purple), olivetolic acid biosynthesis pathway (green), peroxisomal β-oxidation (gray). Upregulated genes (black) and knocked out genes (red) are illustrated. rBOX—reverse β-oxidation; FAB—fatty acid biosynthesis; CoA—coenzyme A; *Ec*—*Escherichia coli*; *Cn*—*Cupriavidus necator*; *Ca*—*Clostridium acetobutylicum*; *Td*—Treponema denticola; *Cs*—*Cannabis sativa*; *gpd2*—glycerol 3-phosphate dehydrogenase 2, *adh1-5*—alcohol dehydrogenase 1–5; *bktB*—β-ketothiolase; *paaH1*—3-hydroxyacyl-CoA dehydrogenase; *crt*—short-chain enoyl-CoA hydratase; *ter*—trans-2-enoyl-CoA reductase; *faa2*—medium-chain fatty acyl-CoA synthetase; fusFAS*—mutant fused fatty acid synthase; TEs—thioesterases; *OLS*—olivetol synthase; *OAC*—olivetolic acid cyclase

Here, we aim to improve the production of hexanoic acid in *S. cerevisiae* through the FAB and the rBOX pathways by implementing various metabolic and genetic engineering approaches. We selected *S. cerevisiae* due to its suitability as a MCFA producer and its ability to express complex plant-derived biosynthetic pathways such as the cannabinoid biosynthesis pathway [28, 35]. While exploring both routes of MCFA biosynthesis separately and in combination, the selective biosynthesis of hexanoic acid was prioritized. We identify a combination of mutations within the FAS complex to allow the best production of hexanoic acid and combine this with the rBOX pathway in a metabolically optimized strain. Furthermore, hexanoic acid titers were improved by preventing β-oxidation-mediated degradation and by engineering the coenzyme A biosynthesis pathway to increase the supply of coenzyme A. In combination, these optimizations led to a significant increase in hexanoic acid production, reaching up to 120 mg L^−1^ in culture supernatants. Finally, we overexpressed the *C. sativa* genes, *OLS* and *OAC*, allowing for a production of up to 15 mg L^−1^ OA using rBOX-derived hexanoyl-CoA. Figure 1 provides a schematic overview of the engineered metabolic pathways.

## Methods

### Strains, media and cultivation

The *E. coli* strain DH10β was used for amplification and propagation of plasmids during cloning and cultivated by standard procedures. For selection, either 100 µg L^−1^ carbenicillin, 50 µg L^−1^ kanamycin or 100 µg L^−1^ chloramphenicol were added to media LB media (1% Tryptone, 0.5% NaCl, 0.5% Bacto™ Yeast Extract (BD), pH 7.5). The *S. cerevisiae* strain BY4741* (MATa his3Δ1 leu2Δ0 met15Δ0 ura3Δ0)* was the background of the *FAS*^*WT*^ (SHY24) and *fas*^*null*^ (SHY34) strains and the strain CEN.PK2-1C (*MATa ura3-52 his3-Δ1 leu2-3,112 trp1-289 MAL2-8C SUC2*) was used for cloning and was the background of all other strains. A description of the strains used in this study is found in Table 1. Yeast strains were cultivated in either complex media (2% peptone (Gibco) and 1% Bacto™ Yeast Extract, (BD)) with 2% dextrose (YPD), synthetic complete (SC) media (0.17% g L^−1^ yeast nitrogen base (YNB) without amino acids (BD) and 0.5% ammonium sulfate at pH 6.3 supplemented with a mixture of amino acids and nucleobases) with 2% dextrose (SCD) or synthetic minimal (SM) media (0.17% yeast YNB without amino acids (BD), 0.5% ammonium sulfate and 20 mM monopotassium phosphate at pH 6.3) with 2% dextrose (SMD). For MCFA production, a specific yeast extract (BD, lot Nr.: 6272556) was used as we had observed that this resulted in the production of higher amounts of MCFA compared to other batches. For selection in complex media, either 200 µg L^−1^ geneticin, 100 µg L^−1^ nourseothricin sulfate or 200 µg L^−1^ hygromycin B was added. To complement the strain auxotrophies, synthetic media was supplemented with 0.171 mM uracil, 0.124 mM histidine, 0.093 mM tryptophan and 0.439 mM leucine. Uracil was omitted in selective synthetic media. For pantothenate-free SMD, pantothenate-free YNB (Sunrise Science Products, Knoxville, TN, USA Cat#: 1512-050) was used. Calcium pantothenate was prepared to working concentrations in water and sterile filtered using 0.22-µm CME filters before being supplemented to the sterilized cultivation media. For solid media, 2% agar–agar was added. Yeast strains were grown at 30 °C and liquid cultures were incubated in shake flasks at 180 rpm. For MCFA production in YPD or SCD, the media was buffered with 100 mM potassium phosphate buffer adjusted to pH 6.5 (KPi). Precultures were grown to the exponential phase prior to being washed and diluted in fresh media to an OD_600_ of 0.1.

**Table 1:** List of strains constructed and used in this study

Strain stock code	Strain name	Relevant description	Source
*S. cerevisiae*			
SHY24	*FAS*^*WT*^	*MATa; his3Δ1; leu2Δ0; met15Δ0; ura3Δ0; ∆faa2*	[36]
SHY34	*fas*^*null*^	**SHY24*** ∆fas1; ∆fas2*	[36]
GDY27	GDY27	* MATa his3-Δ1 leu2-3,112 trp1-289 MAL2-8C* *SUC2; adh1∆::loxP; adh2∆::LEU2; adh3∆::loxP; adh4∆::loxP; adh5∆::loxP; ∆gpd2; ura3∆::P*_*HHF1*_*-*^*Cn*^*bktB-tENO1, P*_*CCW12*_*-*^*Cn*^*paaH1-tIDP1, P*_*ENO2*_*-*^*Ca*^*crt-tPGK1, P*_*TDH3*_*-*^*Td*^*ter-tADH1, kanMX4*	[25]
KSY13	*y*rBOX1	GDY27* ∆faa2*	This study
KSY23	*adh6∆::*^*Ec*^*coaA*	***y*rBOX1*** adh6∆::P*_*PGK1*_*-*^*Ec*^*coaA-tSSA1*	This study
KSY22	*y*rBOX2	***y*rBOX1*** leu2∆::P*_*PGK1*_-^*Ec*^*coaA-tSSA1, natNT2*	This study
KSY26	*y*rBOX3	***y*rBOX2*** P*_*fms1*_*∆::P*_*ADH1*_	This study
*E. coli*			
DH10ß		F– *mcrA* Δ(*mrr-hsdRMS-mcrBC*) φ80*lacZ*ΔM15 Δ*lacX74 recA1 endA1 araD139* Δ (*ara-leu*)7697 *galU galK* λ– *rpsL*(Str^R^) *nupG*	New England Biolabs

The bold represents that the genotype of that strain is identical to the strain

### Plasmid construction and transformation

Genetic elements were amplified from the host genome or from existing plasmids via polymerase chain reaction (PCR) and cloned using either a yeast toolkit (YTK) for modular, multipart assembly based on the golden gate cloning method [38] or via homologous recombination in *S. cerevisiae* [39]. Unless otherwise specified, genetic elements used for cloning via golden gate were derived from the YTK purchased from addgene [38]. A full list of the plasmids and their description can be found in Table 2 and oligonucleotides used for plasmid construction are listed in Table 3. Heterologous genes were codon optimized according to the yeast glycolytic codon usage [40]. Briefly, mutations were incorporated into the fused fatty acid synthase (*fusFAS*) construct via PCR using mutagenic primers and assembled via homologous recombination in using the *fusFAS*^*WT*^ construct (FWV132) as a template [36]. The *hphNT1* cassette in the *fusFAS*^*IAGSMWFY*^ plasmid (ALSV11) was replaced with a *kanMX4* cassette (KSV30). The *kanMX4* cassette was amplified from the pRS41K plasmid and inserted into a cut site within *hphNT1* through homologous recombination in *S. cerevisiae*. The *HIS3* marker in pRS313 was exchanged with a *kanMX4* cassette in the same way to generate an empty vector (KSV48). All other plasmids were cloned using the golden gate cloning method. The *E. coli* pantothenate gene (^*Ec*^*coaA*) was amplified from the plasmid pVS5_4 [41] and cloned via golden gate cloning into a *2µ* expression plasmid (KSV52) or an integrational plasmid with a downstream *natNT2* resistance marker (KSV53). The *ADH1* and *HSP26* promoters and the *FMS1* gene were amplified from CEN.PK2-1C genomic DNA and cloned into *2µ* expression plasmids via golden gate cloning (KSV66, KSV67 and KSV68). The *C. sativa* genes *OLS* and *OAC* were purchased as synthetic sequences from Twist Bioscience, CA, USA, and cloned into a *2µ* expression via golden gate cloning (KSV74). All yeast transformations were performed according to the protocol described by Gietz and Schiestl [42].

**Table 2:** List of plasmids constructed and used in this study

Plasmid stock code	Plasmid name	Relevant description	Source
pRS313	pRS313	*CEN4ARS6, Amp*^*R*^*, HIS3*	Addgene
FWV132	pRS313-*fusFAS*^*WT*^	*CEN4ARS6, Amp*^*R*^*, hphNT1, P*_*TDH3*_*-FAS1-FAS2-tFAS2*	[36]
ALSV7	pRS313-*fusFAS*^*RK*^	*CEN4ARS6, Amp*^*R*^*, hphNT1, P*_*TDH3*_*-FAS1*^*RK*^*-FAS2-tFAS2*	Lab stock
ALSV9	pRS313-*fusFAS*^*RKFY*^	*CEN4ARS6, Amp*^*R*^*, hphNT1, P*_*TDH3*_*-FAS1*^*RK*^*-FAS2*^*FY*^*-tFAS2*	Lab stock
ALSV11	pRS313-*fusFAS*^*IAGSMWFY*^	*CEN4ARS6, Amp*^*R*^*, hphNT1, P*_*TDH3*_*-FAS1*^*IA*^*-FAS2*^*GSMWFY*^*-tFAS2*	Lab stock
ALSV13	pRS313-*fusFAS*^*IARKGSMWFY*^	*CEN4ARS6, Amp*^*R*^*, hphNT1, P*_*TDH3*_*-FAS1*^*IARK*^*-FAS2*^*GSMWFY*^*-tFAS2*	Lab stock
KSV8	pRS313-*fusFAS*^*IAGS*^	*CEN4ARS6, Amp*^*R*^*, hphNT1, P*_*TDH3*_*-FAS1*^*IA*^*-FAS2*^*GS*^*-tFAS2*	This study
KSV9	pRS313-*fusFAS*^*IARKGS*^	*CEN4ARS6, Amp*^*R*^*, hphNT1, P*_*TDH3*_*-FAS1*^*IARK*^*-FAS2*^*GS*^*-tFAS2*	This study
KSV10	pRS313-*fusFAS*^*IAGSMW*^	*CEN4ARS6, Amp*^*R*^*, hphNT1, P*_*TDH3*_*-FAS1*^*IA*^*-FAS2*^*GSMW*^*-tFAS2*	This study
KSV30	pRS313-*fusFAS*^*IAGSMWFY*^	*CEN4ARS6, Amp*^*R*^*, kanMX4, P*_*TDH3*_*-FAS1*^*IA*^*-FAS2*^*GSMWFY*^*-tFAS2*	This study
KSV55	pRCC-N-*ADH6*	*2µ, Amp*^*R*^*, natNT2, P*_*ROX3*_*-*^*Sp*^*cas9-tCYC1, pSNR52-[ADH6]-sgRNA-tSUB4*	This study
SHV42	pRCC-N-*FAA2*	*2µ, Amp*^*R*^*, natNT2, P*_*ROX3*_*-*^*Sp*^*cas9-tCYC1, pSNR52-[FAA2]-sgRNA-tSUB4*	[3]
KSV75	pRCC-H-*P*_*FMS1*_	*2µ, Amp*^*R*^*, hphNT1, P*_*ROX3*_*-*^*Sp*^*cas9-tCYC1, pSNR52-[P*_*FMS1*_*]-sgRNA-tSUB4*	This study
pVS5_4	^*Ec*^*coaA* template	*2µ, natNT2, Amp*^*R*^*, *^*Ec*^*coaA*	[24]
KSV52	^*Ec*^*coaA* expression plasmid	*2µ, Kan*^*R*^*, hphNT1, P*_*PGK1*_*-*^*Ec*^*coaA-tSSA1*	This study
KSV53	^*Ec*^*coaA* integration plasmid	*Kan*^*R*^*, LEU2 5’ HR, P*_*PGK1*_*-*^*Ec*^*coaA-tSSA1, natNT2, LEU2 3’ HR*	This study
KSV66	*P*_*ADH1*_-*FMS1*	*2µ, Kan*^*R*^*, URA3, P*_*ADH1*_*-FMS1-tTDH1*	This study
KSV67	*P*_*HSP26*_-*FMS1*	*2µ, Kan*^*R*^*, URA3, P*_*HSP26*_*-FMS1-tTDH1*	This study
KSV68	*P*_*TEF1*_-*FMS1*	*2µ, Kan*^*R*^*, URA3, P*_*TEF1*_*-FMS1-tTDH1*	This study
KSV74	*OLS*-*OAC*	*2µ, Kan*^*R*^*, URA3, P*_*TEF2*_*-*^*Cs*^*OLS-tADH1, P*_*TEF1*_*-*^*Cs*^*OAC-tHXK2*	This study
pRS41H	EV	*CEN4ARS6, Amp*^*R*^*, hphNT1*	[37]
pRS41K	*kanMX4* template	*CEN4ARS6, Amp*^*R*^*, kanMX4*	[37]
KSV48	EV (pRS313-*kanMX4*)	*CEN4ARS6, Amp*^*R*^*, kanMX4*	This study
SiHV005	EV	*2µ, Kan*^*R*^*, URA3*	Lab stock
SiHV010	EV	*2µ, Kan*^*R*^*, hphNT1*	Lab stock

**Table 3:** Oligonucleotides used for plasmid construction and strain engineering

Oligonucleotides	5′ → 3′ sequence	Application
SHP110	TGACTGCGATGATAGGAGG	Forward primer for amplifying ∆*faa2* locus in SHY24
SHP222	GTGCACCAAGTCAAGTTACG	Reverse primer for amplifying ∆*faa2* locus in SHY24
ALSP7	GTTGTGTTCTACAAAGGTATGAC	Forward primer for insertion of R1834K mutation in *FAS1*
ALSP8	GTCATACCTTTGTAGAACACAAC	Reverse primer for insertion of R1834K mutation in *FAS1*
ALSP13	GGCAATTACTGTATTATTCTTCGCCGGTGTTCGTTGTTACG	Forward primer for insertion of I306A mutation in *FAS1*
ALSP14	CGTAACAACGAACACCGGCGAAGAATAATACAGTAATTGCC	Reverse primer for insertion of I306A mutation in *FAS1*
ALSP15	GTTCTGGTTCTAGTTGGGGTGGTGTTTC	Forward primer for insertion of G1250S and M1251W mutations in *FAS2*
ALSP16	GAAACACCACCCCAACTAGAACCAGAAC	Reverse primer for insertion of G1250S and M1251W mutations in *FAS2*
KSP20	GTTCTGGTTCTTCTATGGGTGGT	Forward primer for insertion of G1250S in *FAS2*
KSP21	ACCACCCATAGAAGAACCAGAAC	Reverse primer for insertion of G1250S in *FAS2*
KSP37	TTAACTATGCGGCATCAGAGCAGATTGTACTGAGAGTGCACCATCAGCGACATGGAGGCC	Forward primer for amplification of *kanMX4* cassette with overhangs for pRS313 to replace marker (*hphNT1* or *HIS3*)
KSP38	TCTCCTTACGCATCTGTGCGGTATTTCACACCGCATATGATCCGGACACTGGATGGCGGC	Reverse primer for amplification of *kanMX4* cassette with overhangs for pRS313 to replace marker (*hphNT1* or *HIS3*)
KSP72	GAGGAAGAAATTCAACACAACAACAAGAAAAGCCAAAATCGTGAGTAAGGAAAGAGTGAG	Forward primer for amplifying ^*Ec*^*coaA* cassette from KSV52 with overhangs for *ADH6* integration locus
KSP73	AAAGAAAGGAGCTACATTTATCAAGAGCTTGACAACATAAAATTAAAGTAGCAGTACTTC	Reverse primer for amplifying ^*Ec*^*coaA* cassette from KSV52 with overhangs for *ADH6* integration locus
KSP88	ACGGTTCAATCGCAATTTCTCCGGAAAGTGCAGTAGCAACTGTAGCCCTAGACTTGATAG	Forward primer for amplifying *P*_*ADH1*_ from *S. cerevisiae* genome with overhangs for *P*_*FMS1*_ locus
VSP375	TTTGGCTGGTGAAACTGTATTCATTGTATATGAGATAGTTGATTGTATGC	Reverse primer for amplifying *P*_*ADH1*_ from *S. cerevisiae* genome with overhangs for *P*_*FMS1*_ locus

Underlined sequences represent insertions or substitutions

### Strain engineering

Genetic modifications in yeast strains were engineered based either on the CRISPR–Cas9 mediated method [43] or via antibiotic resistance marker mediated homologous recombination. Oligonucleotides used for strain engineering are listed in Table 3. The deletion of *FAA2*, the deletion of *ADH6* and simultaneous integration of ^*Ec*^*coaA* in its place and the exchanging of the *FMS1* promoter (*P*_*FMS1*_) with the *ADH1* promoter (*P*_*ADH1*_) were achieved using CRISPR-Cas9. Briefly, plasmids containing expression cassettes for the Cas9 protein, a single guide RNA (sgRNA) structure and a resistance marker were used [43]. Either the *natNT2* resistance marker (pRCC-N) or the *hphNT1* resistance marker (pRCC-H) was used. The *FAA2* gene was deleted using the plasmid pRCC-N-*FAA2* [3] and the DNA break was repaired using an 80 nt oligonucleotide homologous to the regions flanking the gene. The protospacer sequences used to target *ADH6* (CTAGGGCCCAAGTCAAACAG) or *P*_*FMS1*_ (GACCAACATGTGGTAAGGTG) were cloned directly upstream of the sgRNA structure using golden gate cloning, generating the plasmids pRCC-N-*ADH6* and pRCC-H-*P*_*FMS1*_, respectively. The CRISPR–Cas9 plasmid was transformed into the yeast strain together with the corresponding genetic element which was to be integrated into the genome. These elements were amplified using primers containing 30–40 nt homologous overhangs flanking the genetic element to be replaced. Genomic exchange of *P*_*FMS1*_ was achieved by replacing the 500 nt immediately upstream of the start codon with *P*_*ADH1*_. Alternatively, the ^*Ec*^*coaA* gene and was inserted into the *leu2-3,112* locus of the genome via homologous recombination through transformation with the ^*Ec*^*coaA* integrational plasmid (KSV53).

### High-performance liquid chromatography

High-performance liquid chromatography (HPLC) was used to analyze glucose consumption and the production of ethanol, OA and olivetol (OL). For glucose and ethanol measurements, 450 µL media was separated from the cells via centrifugation (15,000 rcf, 5 min) and 50 µL 50% (w/v) 5-sulfosalicylic acid was added. The solution was vortexed, centrifuged (15,000 rcf, 5 min) and the supernatant was transferred to autosampler vials. Samples were analyzed in a UHPLC + system by Thermo Scientific (Dionex UltiMate 3000) equipped with a NUCLEOGEL SUGAR 810 H column (300 × 7.8 mm, 8–10 µm) and a refractive index detector (Thermo Shodex RI-101). The HPLC was operated at 30 °C and 0.5 mM sulfuric acid was used as the mobile phase at flow rate of 0.600 mL min^−1^. OA and OL were extracted by mixing 300 µL cell culture with an ice-cold mixture of 870 µL acetonitrile and 30 µL formic acid. Cells were mechanically lysed using glass beads and vigorous shaking at 4 °C. The samples were then centrifuged (15,000 rcf, 30 min, 4 °C) and filtered into autosampler vials using 0.2 µm nylon filters. The samples were analyzed in a UHPLC + system by Thermo Scientific (Dionex UltiMate 3000) equipped with an Agilent InfinityLab Poroshell 120 EC-C18 column (2.1 × 100 mm, 2.7 µm) and a UV detector (Dionex UltiMate 3000 RS Variable Wavelength Detector) which was operated at 40 °C. A mobile phase consisting of 0.1% (v/v) formic acid in water (solvent A) and acetonitrile (solvent B) was used at a constant flow rate of 0.600 mL min^−1^. 5 µL of sample were injected into the column and analytes were separated using a gradient starting with 70% solvent A and 30% solvent B which was held for 1.5 min. Next, solvent B was linearly increased to 100% in 8 min, linearly decreased to 30% in 0.5 min and held for 1 min. OA and OL were measured using a wavelength of 225 nm and identification and quantification were achieved using real standards. OA standard was purchased from Sigma-Aldrich, Germany (Lot. Nr.: A318844; AmBeed, IL, USA) and OL standard was chemically synthesized within our laboratory and its structure was confirmed via mass spectrometry and nuclear magnetic resonance.

### Fatty acid extraction and derivatization

Fatty acids (FAs) were extracted from the media as described by Henritzi et al*.* [3] and derivatized to fatty acid methyl esters (FAMEs) for gas chromatography (GC) analysis as described by Legras et al*.* [44]. In brief, 10 mL of the culture supernatant were separated via centrifugation (3000 rcf, 15 min) and 0.02 g L^−1^ heptanoic acid (Sigma-Aldrich, Germany, #75190) was added as an internal standard. FAs were then extracted by adding 1 mL 1 M hydrochloric acid (HCl) and 2.5 mL of a 1:1 solution of chloroform and methanol. The phases were mixed through vigorous shaking and then separated via centrifugation (3000 rcf, 10 min). The chloroform phase containing the fatty acids was then transferred to fresh 1.5 mL microcentrifuge tubes and evaporated using a vacuum concentrator (Eppendorf Concentrator 5301) at 60 °C. Next, the FAs were resuspended in 200 µL toluol and transferred to a solution containing 1.5 mL methanol and 300 µL 8% (v/v) HCl solution in methanol. The solution was incubated for 3 h at 100 °C and the FAMEs were extracted by adding 1 mL hexane and 1 mL distilled water and mixing the phases through vigorous shaking. The hexane phase was then transferred to 2 mL autosampler vials for GC analysis.

### Gas chromatography

FAMEs were measured via gas chromatography (GC) using a PerkinElmer Clarus 400 GC equipped with an Elite-5ms Capillary Column (30 m × 0.25 mm I.D. × 0.25 µm, Perkin Elmer, Germany) and a flame ionization detector (FID; Perkin Elmer, Germany). 1 µL sample was injected into the column and split 1:20 using helium as the carrier gas (90 kPa) and an injection temperature of 250 °C. Separation of the FAMEs was achieved using the following temperature program: 5 min hold at 50 °C then increase to 120 °C at 10 °C/min. 5 min hold at 120 °C then increase to 220 °C at 15 °C/min. 10 min hold at 220 °C then increase to 300 °C at 20°/min. Final 5 min hold and cool to 50 °C. Detection occurred at a temperature of 300 °C. Identification and quantification was achieved using hexanoic acid, heptanoic acid, octanoic acid and decanoic acids as standards (Sigma-Aldrich, Germany) which were derivatized to FAMEs using the same protocol.

### Statistical analysis

All experiments were carried out as biological duplicates or triplicates and represented as the mean ± standard deviation (s.d.). The two-tailed unpaired *t*-test was used to analyze statistical significance and was performed using GraphPad Prism (v10.2.0).

## Results and discussion

### Screening of engineered fatty acid synthases for hexanoyl-CoA biosynthesis

To screen for the most efficient engineered FAS construct for the production of hexanoyl-CoA in *S. cerevisiae*, two aspects were considered. In addition to identifying the construct which produced the highest titer of hexanoic acid, the degree of specificity was taken into consideration by monitoring the production of octanoic acid and decanoic acid. We applied a construct in which the *FAS1* and *FAS2* genes were fused to produce a single FAS fusion protein (*fusFAS*), as it had been shown that this fusion allowed for an improved FA production efficiency, presumably due to a quicker and more efficient assembly of the protein complex [36, 45]. Various combinations of amino acid substitutions, previously identified to be involved in chain length control [16], were incorporated into the *fusFAS* construct and cloned under the control of the strong glycolytic *TDH3* promoter (*P*_*TDH3*_). These mutations lie within the three key catalytic domains responsible for chain length control and thus promote the premature release of the growing acyl chain from the FAS complex. The locations of the mutations are within the acyl transferase (AT) domain (I306A), the malonyl-/palmitoyl-CoA transferase (MPT) domain (R1834K) and the ketoacyl synthase (KS) domain (G1250S, M1251W, F1279Y). The AT and MPT domains lie within the FAS β-subunit (Fas1p), whereas the KS domain is embedded within the α-subunit (Fas2p). The mutant *fusFAS* constructs were expressed in the strains SHY24 and SHY34 [36]. SHY24 contains the WT *FAS1* and *FAS2* genes (*FAS*^*WT*^) while SHY34 contains ∆*fas1* and ∆*fas2* mutations (*fas*^*null*^) to prevent competition for acetyl-CoA and malonyl-CoA. Both strains also carried a ∆*faa2* mutation to prevent the degradation of the MCFAs via peroxisomal β-oxidation [46].

**Fig. 2:**
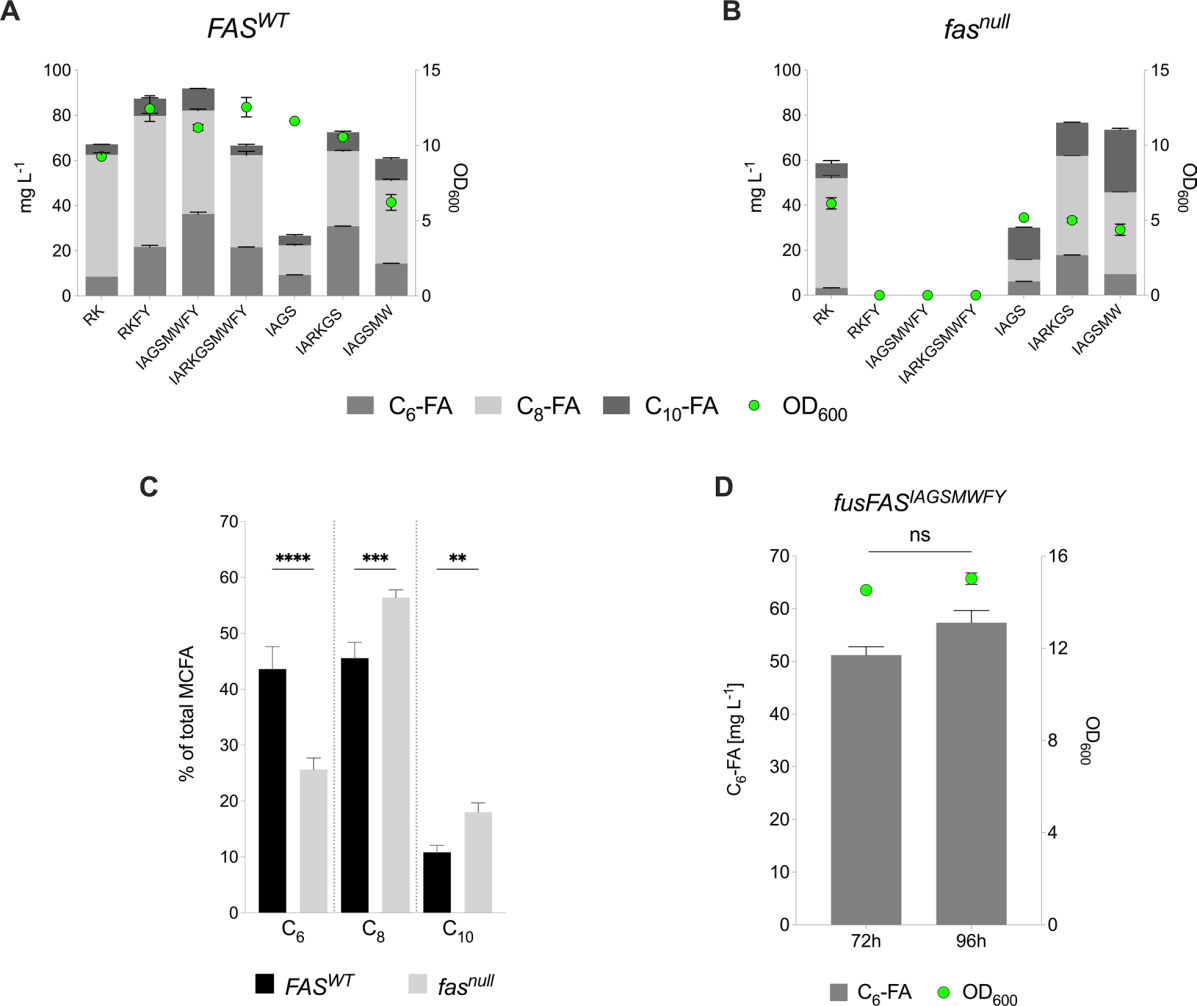
MCFA production using engineered *FAS* constructs in *S. cerevisiae. ***A, B** MCFA output and growth (OD_600_) after 48 h following expression of various mutant fusFAS constructs in a wildtype FAS (*FAS*^*WT*^) or a ∆*fas1* ∆*fas2* (*fas*^null^) strain. **C** The proportion of hexanoic acid (C_6_), octanoic acid (C_8_) and decanoic acid (C_10_) production as a percentage of total MCFA output from the* fusFAS*^*IARKGS*^ construct in a* FAS*^*WT*^ and* fas*^*null*^ strain. **D** C_6_-FA production following expression of the *fusFAS*^*IAGSMWFY*^ construct in a *FAS*^*WT*^ strain after 72 h and 96 h of cultivation. C_6_-FA—hexanoic acid; C_8_-FA—octanoic acid; C_10_-FA—decanoic acid; *fusFAS*—fused fatty acid synthase; OD_600_—optical density at 600 nm. Data represent mean ± s.d.; **A**, **B** and **D** *n* = 2 biologically independent samples. **C** *n* = 4 independent experiments, each representing the mean of two biologically independent samples. Statistical analysis was performed using the two-tailed unpaired *t*-test. *p* > 0.05 = ns (not significant); *p* < 0.05 = *; *p* < 0.01 = **; *p* < 0.001 = ***; *p* < 0.0001 = ****

The production of hexanoic acid was higher for all constructs when expressed in the *FAS*^*WT*^ strain (Fig. 2A) than in the *fas*^*null*^ strain (Fig. 2B). Strikingly, expression of constructs carrying the F1279Y mutation did not allow growth in the *fas*^*null*^ strain (Fig. 2B). Without a source of long chain fatty acids (LCFAs), either via supplementation of oleic acid to the media or expression of a FAS construct capable of producing LCFAs, *fas*^*null*^ mutants are not viable. As the strains expressing the other mutant *fusFAS* constructs were able to grow, we can deduce that these constructs are able to partially complement the *fas*^*null*^ mutation due to a continual low-level synthesis of LCFAs [3, 16, 36, 47]. Although mutant FAS constructs containing the F1279Y mutation have previously been shown to not affect viability [16], we conclude that the combination of mutations tested in this study which included the F1279Y mutation substantially restricts LCFA synthesis, thus rendering the *fas*^*null*^ mutants unviable. Consistent with these findings, three of the best four producers of hexanoic acid in the *FAS*^*WT*^ strain were the constructs which contained the F1279Y mutation, the highest titer reaching 36.37 mg L^−1^ when using the *fusFAS*^*IAGSMWFY*^ construct (Fig. 2A). To exploit this observation, we expressed the *fusFAS*^*IAGSMWFY*^ construct together with the *fusFAS*^*IARKGS*^ construct, as this produced the highest amount of hexanoic acid in the *fas*^*null*^ strain. We hypothesized that the *fusFAS*^*IARKGS*^ construct would complement the ∆*fas1* ∆*fas2* mutation while also maximizing hexanoic acid production (Fig. S1). Although this approach was successful in allowing viability in the *fas*^*null*^ strain, total MCFA output was reduced compared to overexpression of either construct separately. Expressing both constructs together in the *FAS*^*WT*^ strain resulted in a higher MCFA production than in the *fas*^*null*^ strain but was lower than expression of *fusFAS*^*IAGSMWFY*^ alone. Expressing multiple *FAS* constructs in parallel may result in a limitation of precursor supply or reducing power and could therefore explain why we were unable to observe a synergistic or additive effect on MCFA production. An alternative approach to increase hexanoic acid production may be to moderately downregulate the expression of the WT *FAS* genes in order to maximize precursor supply for the mutant *FAS* constructs while still retaining a sufficient degree of fitness.

Moreover, the percentage of hexanoic acid compared to total MCFA output was higher in the *FAS*^*WT*^ strain than *fas*^*null*^ strain, possibly due to the higher pressure for the *fas*^*null*^ mutants to elongate fatty acids and ensure survival. Indeed, analysis of various independent experiments in which the *fusFAS*^*IARKGS*^ construct was expressed in the *FAS*^*WT*^ strain showed that hexanoic acid accounted for 43.6% of the total MCFA output whereas the portion of hexanoic acid produced by the same construct in the *fas*^*null*^ strain amounted to 25.6% (Fig. 2C). Furthermore, the *FAS*^*WT*^ strain displayed improved growth compared to the *fas*^*null*^ strain, as determined by the final OD_600_. The higher production titer of hexanoic acid may therefore also be partly attributed to a larger accumulation of biomass which outweighs the negative implications of competing with the WT FAS for precursor supply. Finally, the cultivation time of the best hexanoic producer (*fusFAS*^*IAGSMWFY*^) was extended from 48 h to 72 h and 96 h in the *FAS*^*WT*^ strain to deduce whether titers would continue to increase (Fig. 2D). Indeed, production continued to increase over time reaching titers of 51.22 ± 1.57 mg L^−1^ after 72 h and 57.35 ± 2.32 mg L^−1^ after 96 h. We therefore continued to work with an extended cultivation time when expressing the mutant *fusFAS* constructs in our strains.

### Optimizing and combining reverse β-oxidation pathway with fatty acid biosynthesis for hexanoyl-CoA biosynthesis

**Fig. 3:**
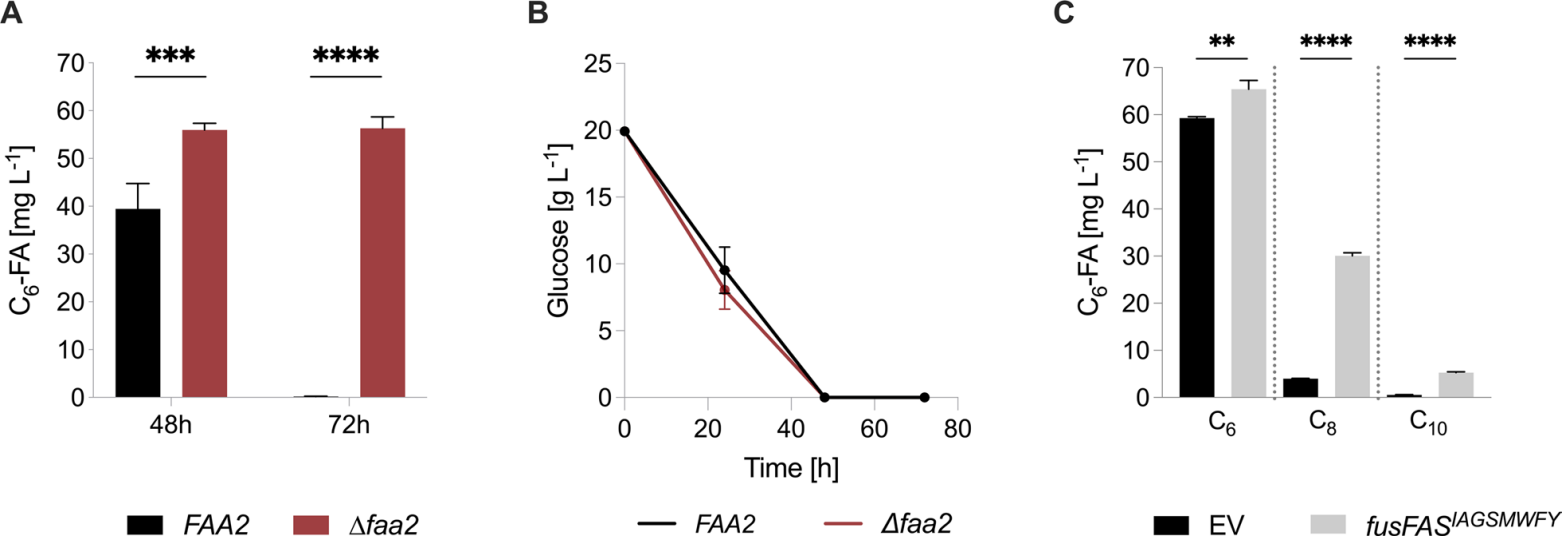
*FAA2* knockout prevents degradation of reverse β-oxidation pathway-derived hexanoic acid in *S. cerevisiae. ***A** Effect of *FAA2* deletion on hexanoic acid degradation after 48 h and 72 h in rBOX pathway strain. **B** Effect of *FAA2* deletion on glucose consumption in rBOX pathway strain. **C** MCFA output after 96 h following expression of *fusFAS*^*IAGSMWFY*^ in the ∆*faa2* rBOX strain (*y*rBOX1). C_6_-FA—hexanoic acid; *fusFAS*—fused fatty acid synthase; rBOX—reverse β-oxidation. Data represent mean ± s.d.; *n* = 3 biologically independent samples. Statistical analysis was performed using the two-tailed unpaired *t*-test. *p* > 0.05 = ns (not significant); *p* < 0.05 = *; *p* < 0.01 = **; *p* < 0.001 = ***; *p* < 0.0001 = ****

Alternatively, we sought to implement a heterologous reverse β-oxidation (rBOX) pathway using enzymes derived from multiple organisms for the production of hexanoic acid in *S. cerevisiae* [11, 25, 28]. A β-ketothiolase (*bktB*) and a 3-hydroxyacyl-CoA dehydrogenase (*paaH1*) were derived from *Cupriavidus necator*, a crotonase (*crt*) from *Clostridium acetobutylicum* and a trans-2-enoyl-CoA reductase (*ter*) from *Treponema denticola.* This pathway was chosen primarily for its ability to synthesize hexanoic acid as the principal product while butyric, octanoic and decanoic acid contributed to only a small fraction of the total MCFA output [25]. Moreover, we used the *S. cerevisiae* strain GDY27 in which the rBOX pathway was stably integrated into the *URA3* locus in the genome [25]. The strain had also been engineered to block competing metabolic pathways through knockouts of the alcohol dehydrogenase genes 1 to 5 (∆*adh1-5*) and glycerol 3-phosphate dehydrogenase 2 (∆*gpd2*) to prevent the formation of ethanol and glycerol during glucose fermentation, as this had been previously shown to increase product output from the rBOX pathway [24, 25]. Thus, the carbon flux is redirected towards the synthesis of cytosolic acetyl-CoA and reducing power in the form of NADH is preserved. In order to stabilize the production of hexanoic acid, we knocked out the *FAA2* gene in GDY27, generating the strain *y*rBOX1 (∆*faa2*). This prevented MCFA degradation and therefore increased hexanoic acid production compared to the parent strain (Fig. 3A). The GDY27 strain (*FAA2*) produced 39.44 ± 5.31 mg L^−1^ hexanoic acid after 48 h and this was almost fully consumed by 72 h. In contrast, *y*rBOX1 (∆*faa2*) produced 55.98 ± 1.37mg L^−1^ after 48 h which remained stable until 72 h. Moreover, glucose was fully consumed between 24 and 48 h (Fig. 3B). These results suggest that the *FAA2* strain began to reuptake and degrade the accumulated hexanoic acid via the β-oxidation pathway after 48 h and did not continue to produce hexanoic acid once glucose was depleted. In contrast, hexanoic acid was not consumed in the ∆*faa2* strain and levels remained consistent until 72 h.

Next, to further increase hexanoic acid biosynthesis, we overexpressed the *fusFAS*^*IAGSMWFY*^ construct (ALSV11) in the ∆*faa2* rBOX strain (*y*rBOX1) with the rationale for prolonging the time of MCFAs synthesis as each pathway is active during different growth phases. The previous results suggest that the rBOX pathway is primarily active during glucose consumption, whereas the main production of MCFAs via the mutant FAB pathway begins once glucose is depleted. After 96 h of cultivation in YPD, 59.3 ± 0.29 mg L^−1^ hexanoic acid was measured from the rBOX pathway alone (EV) which was increased by approximately 10% to 65.4 ± 1.8 mg L^−1^ when the *fusFAS*^*IAGSMWFY*^ construct was expressed (Fig. 3C). Furthermore, combining the mutant FAB and rBOX pathways resulted in a 7.5-fold and tenfold increase in octanoic and decanoic acid production, respectively. Thus, the total output of MCFAs was increased by approximately 58% when combining the two pathways.

### Increasing coenzyme A supply to improve hexanoyl-CoA biosynthesis from the reverse β-oxidation pathway

**Fig. 4:**
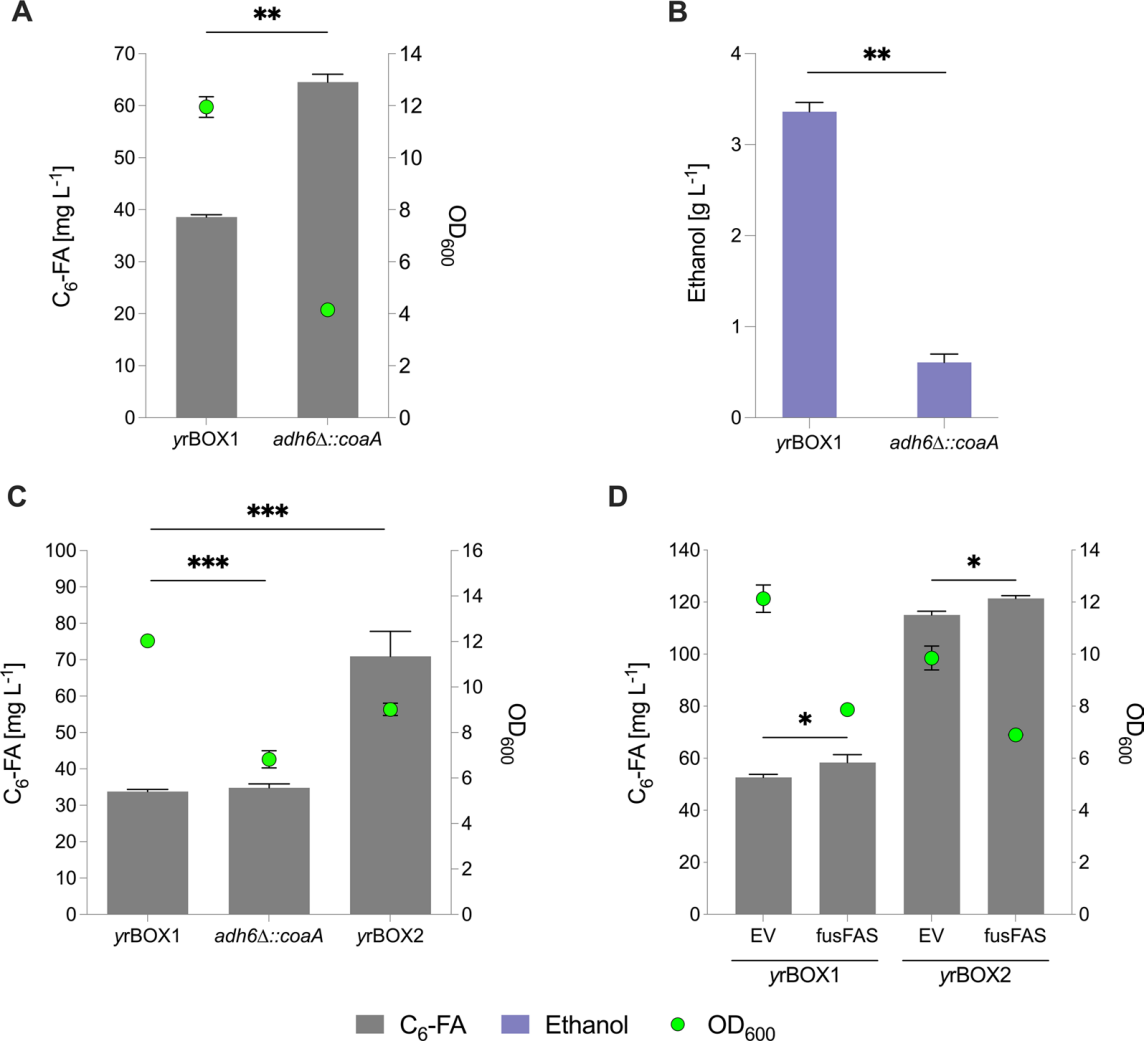
Increasing CoA biosynthesis for hexanoic acid production in *S. cerevisiae. ***A** C_6_-FA production and growth (OD_600_) after 96 h following integration and overexpression of an *E. coli*-derived pantothenate kinase (^*coaAEc*^) in the *ADH6* locus (KSY23; *adh6∆::coaA*) compared to parent strain (*y*rBOX1). **B** Ethanol production in *adh6∆::coaA* strain after 96 h. **C** C_6_-FA production and growth (OD_600_) after 48 h following integration of ^*coaAEc*^ in the* leu2-3,112* locus (*y*rBOX2) or in the *ADH6* locus (KSY23; *adh6∆::coaA*) compared to parent strain (*y*rBOX1). **D** C_6_-FA production and growth (OD_600_) after 96 h following overexpression of *fusFAS*^*IAGSMWFY*^ construct (ALSV11) in *y*rBOX1 and *y*rBOX2 strain with pRS41H as an EV control. CoA—coenzyme A; C_6_-FA—hexanoic acid; OD_600_—optical density at 600 nm; *fusFAS*—mutant fused fatty acid synthase; EV—empty vector. Data represent mean ± s.d.; **A**, **B** *n* = 2 biologically independent samples **C**, **D** *n* = 3 biologically independent samples. Statistical analysis was performed using the two-tailed unpaired *t*-test. *p* > 0.05 = ns (not significant); *p* < 0.05 = *; *p* < 0.01 = **; *p* < 0.001 = ***; *p* < 0.0001 = ****

Further to increasing MCFA synthesis by combing both the rBOX and FAB metabolic pathways in a single recombinant strain, we aimed to increase the synthesis of hexanoic acid alone as an indirect indicator of hexanoyl-CoA synthesis due to its relevance as a precursor for cannabinoid biosynthesis. Although co-expression of the rBOX pathway and a mutant *fusFAS* resulted in an increase in hexanoic acid titer (Fig. 3C), we decided to proceed by optimizing the rBOX pathway alone due to the high specificity of hexanoic acid production. Intracellular coenzyme A (CoA) levels were found to be increased in *E. coli* when the rate limiting enzyme in CoA biosynthesis, pantothenate kinase (^*Ec*^*coaA*), was overexpressed together with supplementation of pantothenic acid to the culture [48]. As the output of the rBOX pathway is dependent on the level of cytosolic acetyl-CoA, we aimed to increase CoA biosynthesis in our strain. Indeed, the availability of free CoA was shown to limit the production of n-butanol derived from the expression of a heterologous rBOX pathway in *S. cerevisiae* [41]. Here, the overexpression of ^*Ec*^*coaA* led to an increase in n-butanol production and additional supplementation of pantothenate to the media further increased production. We therefore cloned and overexpressed a codon optimized version of ^*Ec*^*coaA* (KSV52) in *y*rBOX1 which resulted in a production of 62.71 ± 4.28 mg L^−1^ hexanoic acid, corresponding to 1.6-fold increase compared to an EV control (Fig. S2). Next, we seamlessly and stably integrated ^*Ec*^*coaA* under the control of the strong 3-phosphoglycerate kinase 1 promoter (*P*_*PGK1*_) via CRISPR–Cas9. We chose to replace the *ADH6* gene in the *y*rBOX1 strain with ^*Ec*^*coaA* in a two-pronged effort to further increase metabolic flux toward acetyl-CoA biosynthesis by lowering ethanol production, based on a previous approach [24]. We found that the *adh6*∆::*coaA y*rBOX1 strain (KSY23) increased hexanoic acid production by 67% reaching a titer of 64.5 ± 1.5 mg L^−1^ after 96 h of cultivation in complex media compared to 38.6 ± 0.4 mg L^−1^ in the parent *y*rBOX1 strain (Fig. 4A). Nevertheless, significant growth and production only began after 48 h of cultivation in the *adh6*∆::*coaA* strain, presumably due to the reduced ability for the cells to rapidly ferment glucose in the absence of alcohol dehydrogenases. This was reflected in both the total biomass accumulation, reaching an OD_600_ of only 4.2 compared to 12.0 in the parent strain (Fig. 4A), and in the lower ethanol production, reaching 0.61 ± 0.1 g L^−1^ in contrast to 3.36 ± 0.1 g L^−1^ (Fig. 4B). As a reduced fitness and longer cultivation times are industrially undesirable, we stably integrated ^*Ec*^*coaA* into the redundant *leu2-3,112* locus of *y*rBOX1 through homologous recombination, thereby leaving the *ADH6* gene intact. We found that the resultant strain (*y*rBOX2) produced 71.0 ± 6.9 mg L^−1^ hexanoic acid after 48 h of cultivation in complex media, corresponding to a 2.1-fold increase over the parent *y*rBOX1 strain (33.8 ± 0.6 mg L^−1^) and a twofold increase over the *adh6*∆::*coaA* strain (34.8 ± 1.1 mg L^−1^; Fig. 4C). Thus, in addition to exhibiting a superior production, the growth of *y*rBOX2 was only slightly perturbed compared to the parent strain, likely due to the toxicity of hexanoic acid accumulation, and the cultivation time remained at 48 h in contrast to the *adh6*∆::*coaA* strain.

As the precursors of FAS mediated fatty acid biosynthesis are acetyl-CoA and malonyl-CoA, it was speculated that the lower hexanoic acid titers derived from the *fusFAS*^*IAGSMWFY*^ construct when expressed in combination with the rBOX pathway (approx. 6 mg L^−1^; Fig. 3C) compared to expression of the *fusFAS*^*IAGSMWFY*^ alone (approx. 57 mg L^−1^; Fig. 2D) may have been due to a limited CoA supply. We therefore hypothesized that combining the two pathways in the ^*Ec*^*coaA* overexpressing strain may result in an increased production of hexanoic acid due to the greater supply of CoA. For this, the strains *y*rBOX1 and *y*rBOX2 were transformed with the *fusFAS*^*IAGSMWFY*^ (ALSV11) construct or an EV control (Fig. 4D). We observed a 9% increase in hexanoic acid production by the *y*rBOX1 strain when *fusFAS*^*IAGSMWFY*^ was expressed compared to the EV control, reaching 59.4 ± 1.7 mg L^−1^. As expected, the overall production in *y*rBOX2 was approximately 2.1-fold higher compared to *y*rBOX1, reaching 117.6 ± 2.0 mg L^−1^ in the EV control and 122.7 ± 1.7 mg L^−1^ following overexpression of the mutant *FAS* construct. However, the production of hexanoic acid only corresponded to an increase of 4.3% when both pathways were combined in the *y*rBOX2 strain compared to rBOX alone (EV). Moreover, co-expression in *y*rBOX2 resulted in the production of 20.1 ± 0.8 mg L^−1^ octanoic acid and 5.4 ± 0.3 mg L^−1^ decanoic acid in contrast to only 5.6 ± 0.1 mg L^−1^ and 0.7 mg L^−1^, in the EV control, respectively (Fig. S3). As with the previous experiments, we conclude that the advantage of achieving a higher specificity in hexanoic acid production using only the rBOX pathway outweighs the mild increase in production observed when combining the two pathways.

### Increasing pantothenate supply to improve hexanoyl-CoA biosynthesis

**Fig. 5:**
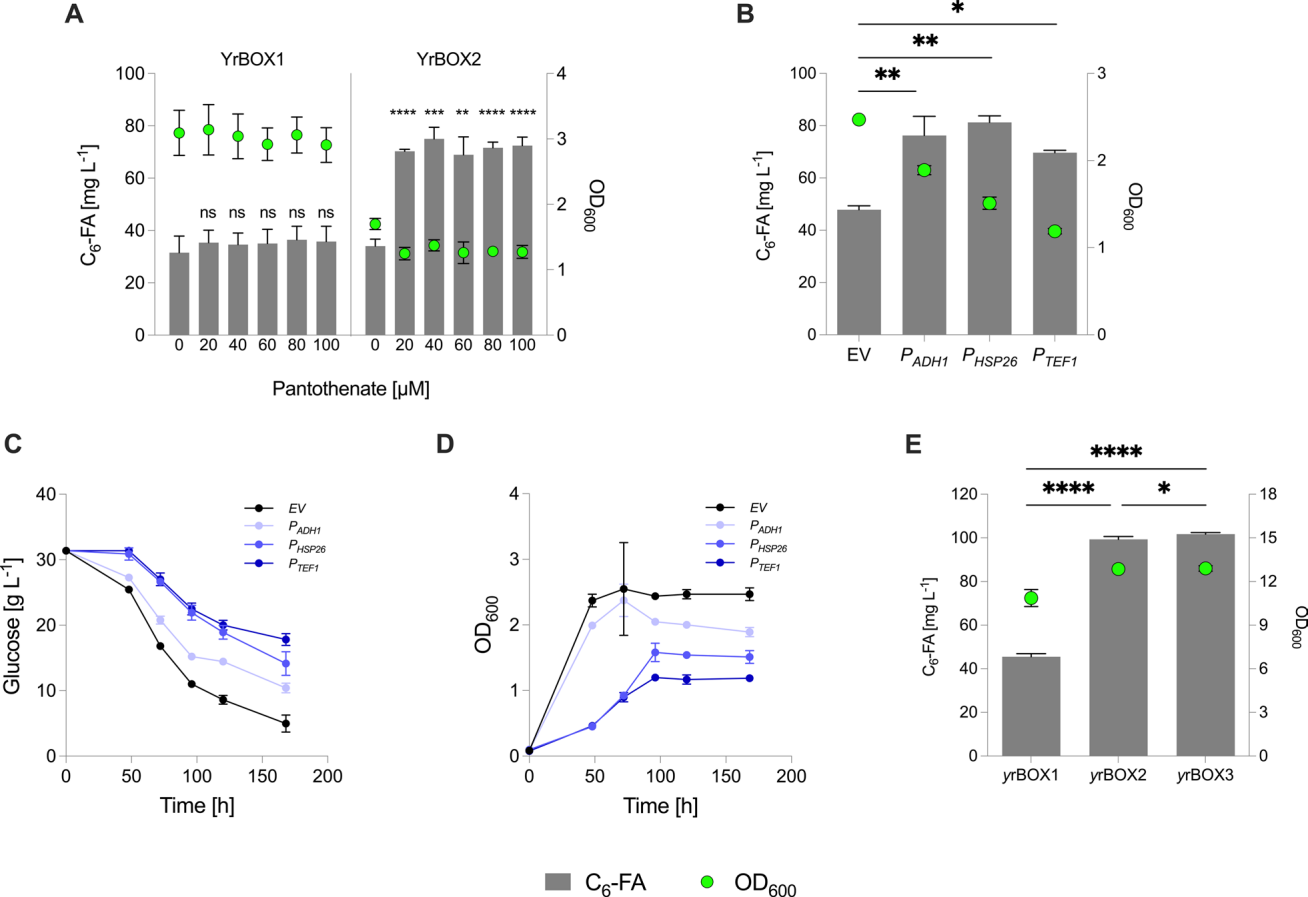
Increasing pantothenate supply for CoA biosynthesis in *S. cerevisiae. ***A** C_6_-FA production and growth (OD_600_) after 168 h of cultivation in pantothenate-free SMD media supplemented with 0, 20, 40, 60, 80 or 100 µM pantothenate in the *y*rBOX1 and *y*rBOX2 strain. **B** C_6_-FA production and growth (OD_600_) after 168 h in selective SCD media following overexpression of *FMS1* under the control of three different promoters (*P*_*ADH1*_,* P*_*HSP26*_,* P*_*TEF1*_) and an EV control in *y*rBOX2. **C** Glucose consumption of *y*rBOX2 expressing *FMS1* constructs. **D** Growth (OD_600_) of *y*rBOX2 expressing *FMS1* constructs. **E** C_6_-FA production and growth (OD_600_) following genomic exchange of the *FMS1* promoter with the *ADH1* promoter (*y*rBOX3) compared to the parent strain (*y*rBOX2) and its parent strain (*y*rBOX1) after 48 h in complex media. CoA—coenzyme A; C_6_-FA—hexanoic acid; OD_600_—optical density at 600 nm; EV—empty vector. Data represent mean ± s.d.; **A**, **E**: *n* = 3 biologically independent samples. **B**–**D**: *n* = 2 biologically independent samples. Statistical analysis was performed using the two-tailed unpaired *t*-test. *p* > 0.05 = ns (not significant); *p* < 0.05 = *; *p* < 0.01 = **; *p* < 0.001 = ***; *p* < 0.0001 = ****

*S. cerevisiae* is able to synthesize pantothenate endogenously from β-alanine and pantoate, catalyzed by Pan6p [49, 50] or take it up from its environment via a transporter encoded by *FEN2* [51]. Therefore, in order to determine whether pantothenate levels were limiting the full effect of overexpressing pantothenate kinase for hexanoic acid production, we performed feeding experiments using increasing concentrations of pantothenic acid. The *y*rBOX1 and *y*rBOX2 strains were cultivated in pantothenate-free SMD media and pantothenate was added at concentrations of 0, 20, 40, 60, 80 or 100 µM (Fig. 5A). With no additional supplementation of pantothenate, the production of hexanoic acid was limited to 31.5 ± 6.3 and 34.1 ± 2.7 mg L^−1^ in *y*rBOX1 and *y*rBOX2, respectively. This signals that overexpression of pantothenate kinase alone does not improve hexanoic acid production. However, supplementation of 20 µM resulted in a twofold increase in production by *y*rBOX2 (70.3 ± 0.8 mg L^−1^) while no significant effect was observed in *y*rBOX1 (35.4 ± 4.7 mg L^−1^). Growth was also affected by ^*Ec*^*coaA* overexpression. Surprisingly, this was also the case for *y*rBOX2 cultures in which pantothenate was not added and hexanoic acid production was similar to *y*rBOX1. This indicates that toxicity resulting from an increase in hexanoic acid production is not the sole cause of biomass reduction in *y*rBOX2. Nevertheless, increased production was also coupled to a mild decrease in biomass when pantothenate was added. Increasing the concentration of pantothenate further had no significant effect on production or growth suggesting that pantothenate kinase or other downstream enzymes involved in CoA biosynthesis may still be limiting production.

In light of these results, we aimed to overproduce pantothenate internally by overexpressing *FMS1* which encodes the rate limiting enzyme involved in β-alanine biosynthesis [49]. For this, *FMS1* was cloned and overexpressed in *y*rBOX2 under the control of one of three different yeast promoters of various strengths and temporal expression regulation (*P*_*ADH1*_, *P*_*HSP26*_ and *P*_*TEF1*_; Fig. 5B). Overexpression of the constructs all resulted in an increase in hexanoic acid production compared to an EV control. Interestingly, the lowest increase in production was observed using *P*_*TEF1*_*-FMS1* resulting in a 45% increase (69.7 ± 0.9 mg L^−1^) although the *TEF1* promoter is reported to show strong expression in yeast when glucose is the carbon source [52] and is widely regarded as a strong promoter under these conditions. *P*_*ADH1*_*-FMS1* increased production by 59% (76.3 ± 7.3 mg L^−1^), consistent with previous reports of increasing CoA synthesis in yeast [41] while *P*_*HSP26*_*-FMS1* led to the highest increase in production of 70% (81.3 ± 2.5 mg L^−1^), despite being described to be weaker than the *TEF1* promoter that is activated during later growth stages on glucose [52]. Moreover, growth (Fig. 5C) and glucose consumption (Fig. 5D) were drastically reduced when using both *P*_*TEF1*_ and *P*_*HSP26*_. *P*_*ADH1*_ consumed glucose and grew at a similar rate to the EV control during earlier stages of cultivation, although overall consumption and growth was reduced. These data provide a further example of the importance of calculating the trade-off between increased production and strain fitness. We therefore continued using *P*_*ADH1*_ to control *FMS1* expression and exchanged the *FSM1* promoter with the *ADH1* promoter within the genome of *y*rBOX2. Surprisingly, the hexanoic acid output of the resultant strain, *y*rBOX3, only increased marginally (101.8 ± 0.6 mg L^−1^) compared to *y*rBOX2 (99.3 ± 1.4 mg L^−1^; Fig. 5E) in complex media. This may be due to pantothenate saturation if the amounts present in complex media are sufficient for CoA biosynthesis, thus masking the benefit of an increased internal pantothenate biosynthesis. Nevertheless, we deduce that it is advantageous to use a strain capable of overproducing pantothenate itself in light of future biotechnological applications in which an external pantothenate supply is undesirable. Moreover, as our previous data indicated a bottleneck in CoA biosynthesis in *y*rBOX2 when supplemented with levels above 20 µM of pantothenate, overexpression of the whole endogenous CoA biosynthesis pathway may be a promising option, as has been reported by Olzhausen and colleagues [53]. Here, they identified a single W331R mutation within the *CAB1* gene to prevent feedback inhibition by acetyl-CoA and found that overexpression of all the genes involved in CoA biosynthesis from pantothenate (*CAB1*-*5*), including the mutant *CAB1*^*W331R*^, resulted in a substantial increase in CoA biosynthesis.

### Production of a key cannabinoid intermediate via the reverse β-oxidation pathway

**Fig. 6:**
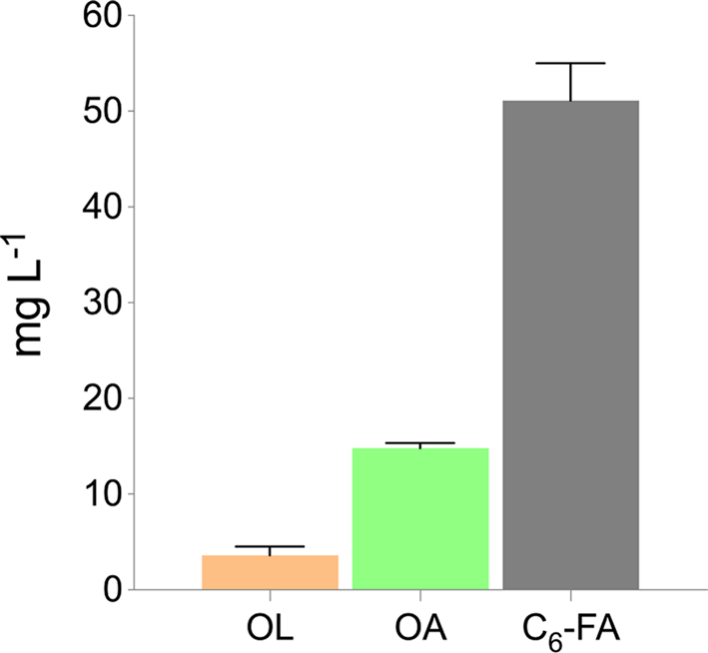
Production of olivetolic acid using reverse β-oxidation-derived hexanoyl-CoA in *S. cerevisiae. *Olivetol (OL), olivetolic acid (OA) and hexanoic acid (C_6_-FA) production following expression of olivetol synthase (*OLS*) and olivetolic acid cyclase (*OAC*) on a multicopy plasmid in *y*rBOX1 cultivated in selective SCD media for 120 h. Data represent mean ± s.d.; *n* = 3 biologically independent samples

To produce olivetolic acid (OA) from de novo synthesis of hexanoyl-CoA, we overexpressed the *C. sativa* genes *OLS* and *OAC* in *y*rBOX1 and cultivated the strains in selective SCD media (Fig. 6). In so doing, we were able to achieve a titer of 14.8 ± 0.5 mg L^−1^ OA. Furthermore, 3.6 ± 0.9 mg L^−1^ olivetol (OL), a side product which forms as a result of a spontaneous decarboxylative aldol condensation reaction of the intermediate produced by OLS in the absence of OAC [54], was measured. This indicates that the expression or the activity of OAC is lower than that of OLS, thus limiting the production of OA. Finally, 51.1 ± 3.9 mg L^−1^ hexanoic acid was measured in the media at the end of the cultivation. This signals that the supply of rBOX-derived hexanoyl-CoA is sufficient for OA synthesis, however, there is a substantial bottleneck in either *OLS* expression or activity. Thus, endogenous TEs are able to hydrolyze hexanoyl-CoA more rapidly than OLS is able to use it as a substrate. Furthermore, despite displaying low-level hexanoyl-CoA ligase activity through an endogenous acyl activating enzyme [28], *S. cerevisiae* lacks an efficient hexanoyl-CoA ligase which may explain the accumulation of large amounts of extracellular hexanoic acid after cultivation. To overcome these problems, stable integration of *OLS* and *OAC* genes into the genome may be beneficial. Although *OLS* and *OAC* were expressed on multicopy (*2µ*) plasmids, integration of a single copy of a gene into the genome can lead to higher production of a desired product. We observed this in the case of pantothenate kinase as overexpression of ^*Ec*^*coaA* on a multicopy plasmid led to an increase in hexanoic acid production of 1.4-fold after 48 h (50.6 ± 3.2 mg L^−1^; Fig. S2), while stable integration of a single copy (*y*rBOX2) led to a 2.1-fold increase in hexanoic acid, reaching 71.0 ± 6.9 mg L^−1^ (Fig. 4C). Furthermore, it may be necessary to integrate multiple copies of *OLS* and *OAC* into the genome as it had also been observed that introducing further copies both genes led to an increase in downstream cannabinoid production in *S. cerevisiae* compared to a single copy integration strain [28, 32]. Moreover, to prevent the wasteful production of OL, it may be necessary to engineer a strain displaying a higher expression of *OAC* compared to *OLS* or to promote close proximity of the enzymes to ensure that the linear tetraketide product of OLS is directly accessible for OAC.

## Conclusion

MCFAs are industrially valuable compounds which have uses ranging from the chemical to the energy sectors. The biosynthesis of these compounds using microbial fermentation processes has been widely investigated and implemented. Nevertheless, combining different metabolic routes in a single recombinant strain has not been investigated to date. Here, we specifically optimize the production of hexanoic acid in *S. cerevisiae* using both the FAB and rBOX pathways independently and in combination. Despite observing an increase in hexanoic acid production when expressing a mutant *FAS* construct in a strain containing a rBOX pathway, we suggest that implementing the rBOX pathway alone is more beneficial when specificity of a single MCFA species is required. However, an increased total MCFA output can be achieved when combining both pathways as these are active in different stages of growth. We therefore demonstrate the feasibility of combining both pathways and propose that the composition of this output can be altered as desired by incorporating different mutations within the FAS complex as described in this work or by applying different enzymes within the rBOX pathway which has been reported elsewhere [25]. Finally, we are able to synthesize the cannabinoid precursor OA by expressing the *C. sativa*-derived genes *OLS* and *OAC* in an optimized hexanoic acid producing strain (*y*rBOX1). Our work therefore also demonstrates the potential to synthesize higher levels OA than previously reported without additional hexanoic acid feeding and provides the groundwork for further optimization of the cannabinoid biosynthesis pathway in *S. cerevisiae*.

## Supplementary Information

Supplementary Figure 1. Combining mutant FAS constructs in a single strain

Supplementary Figure 2. Plasmid-based overexpression of E. coli pantothenate kinase 776 (coaA).

Supplementary Figure 3. Plasmid-based overexpression of mutant fusFAS construct in 774 reverse β-oxidation strains

## Data Availability

No datasets were generated or analysed during the current study.
