# Supplementary figures and images for: Optimizing hexanoic acid biosynthesis in Saccharomyces cerevisiae for the de novo production of olivetolic acid
Source: Biotechnol Biofuels Bioprod. 2024 Dec 4;17:141. doi: 10.1186/s13068-024-02586-2 (PMC11616333; doi:10.1186/s13068-024-02586-2)

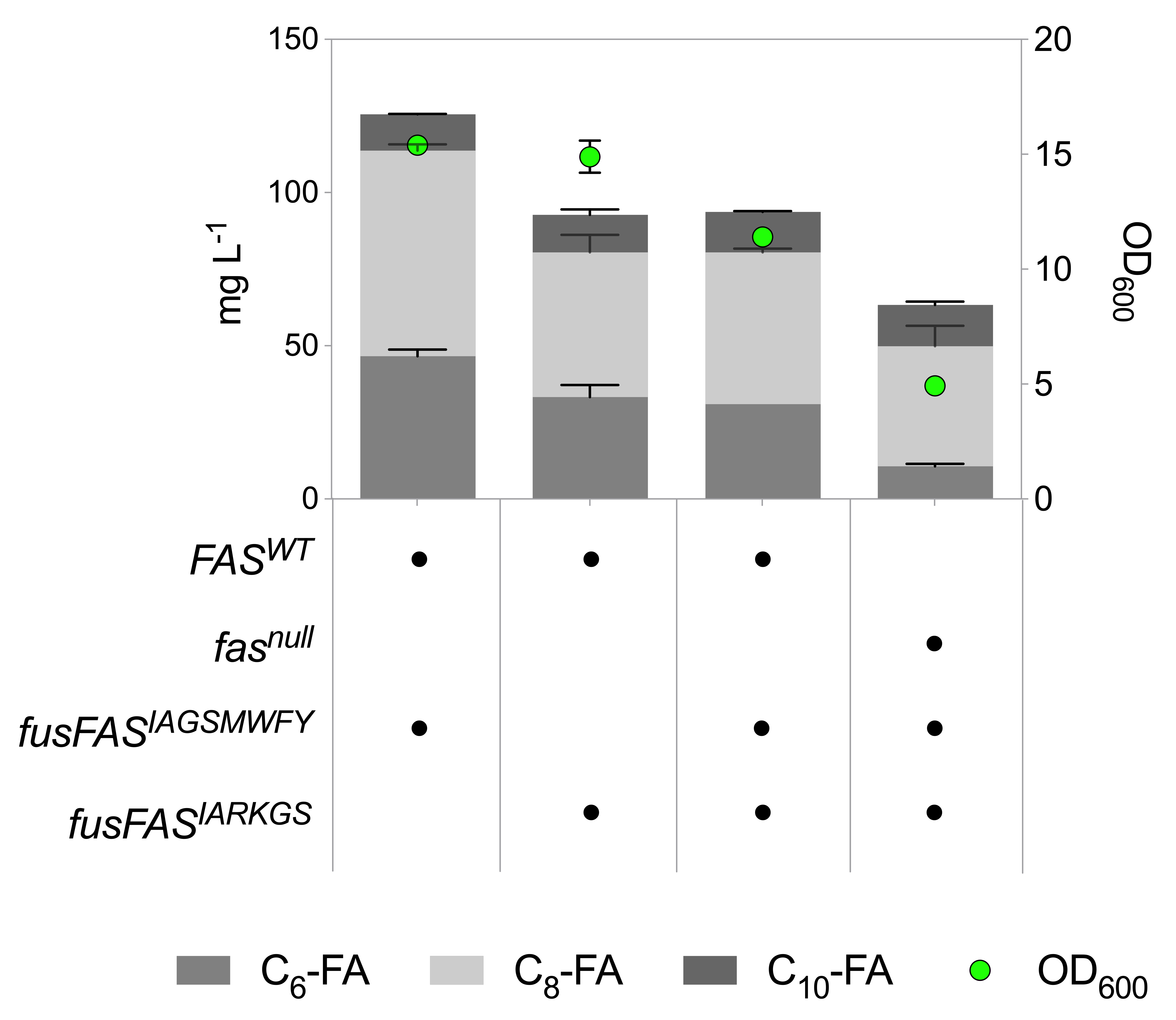

Supplement: Supplementary file 1 — Supplementary Figure 1. Combining mutant FAS constructs in a single strain [file 13068_2024_2586_MOESM1_ESM.tiff]

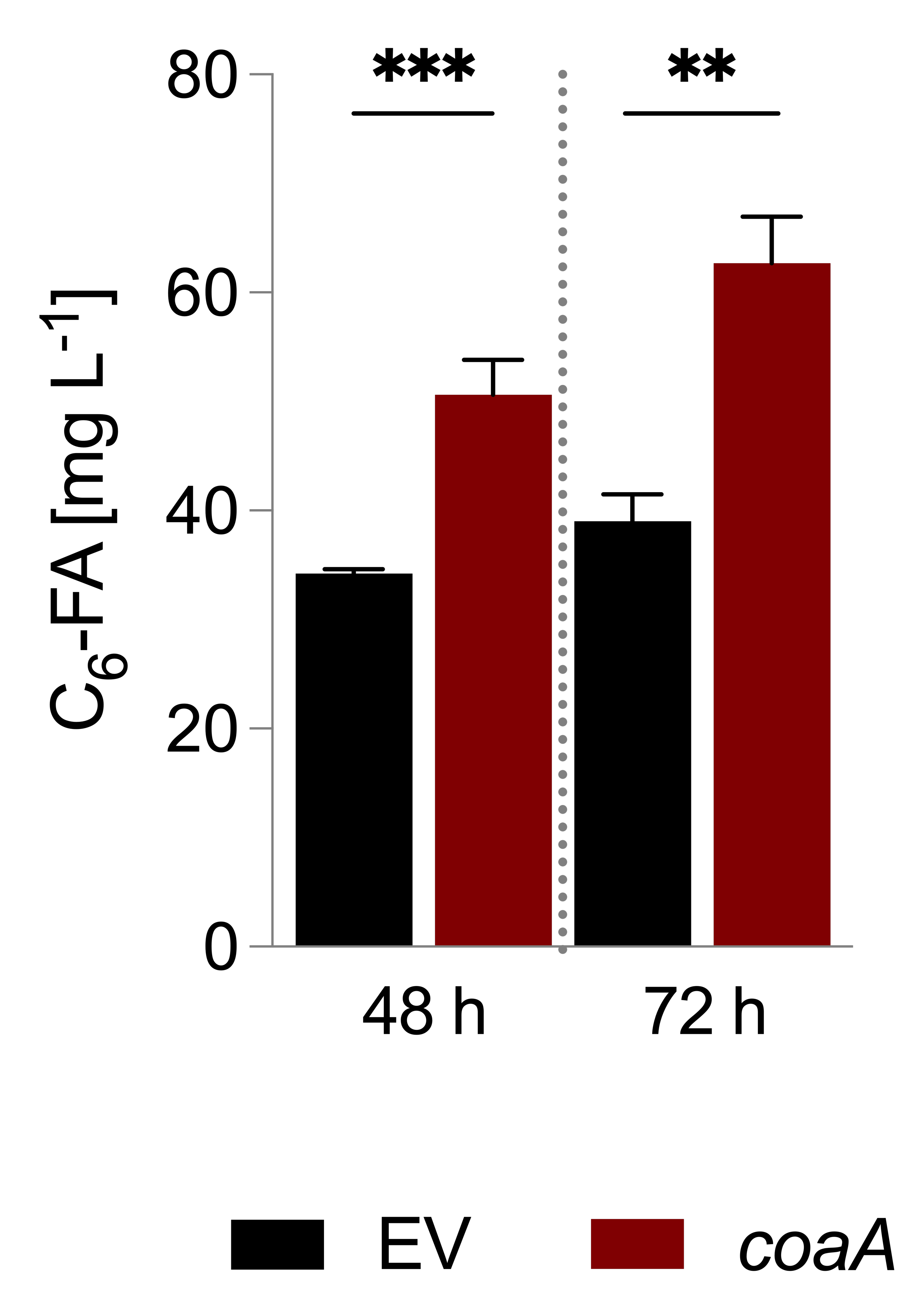

Supplement: Supplementary file 2 — Supplementary Figure 2. Plasmid-based overexpression of E. coli pantothenate kinase 776 (coaA). [file 13068_2024_2586_MOESM2_ESM.tiff]

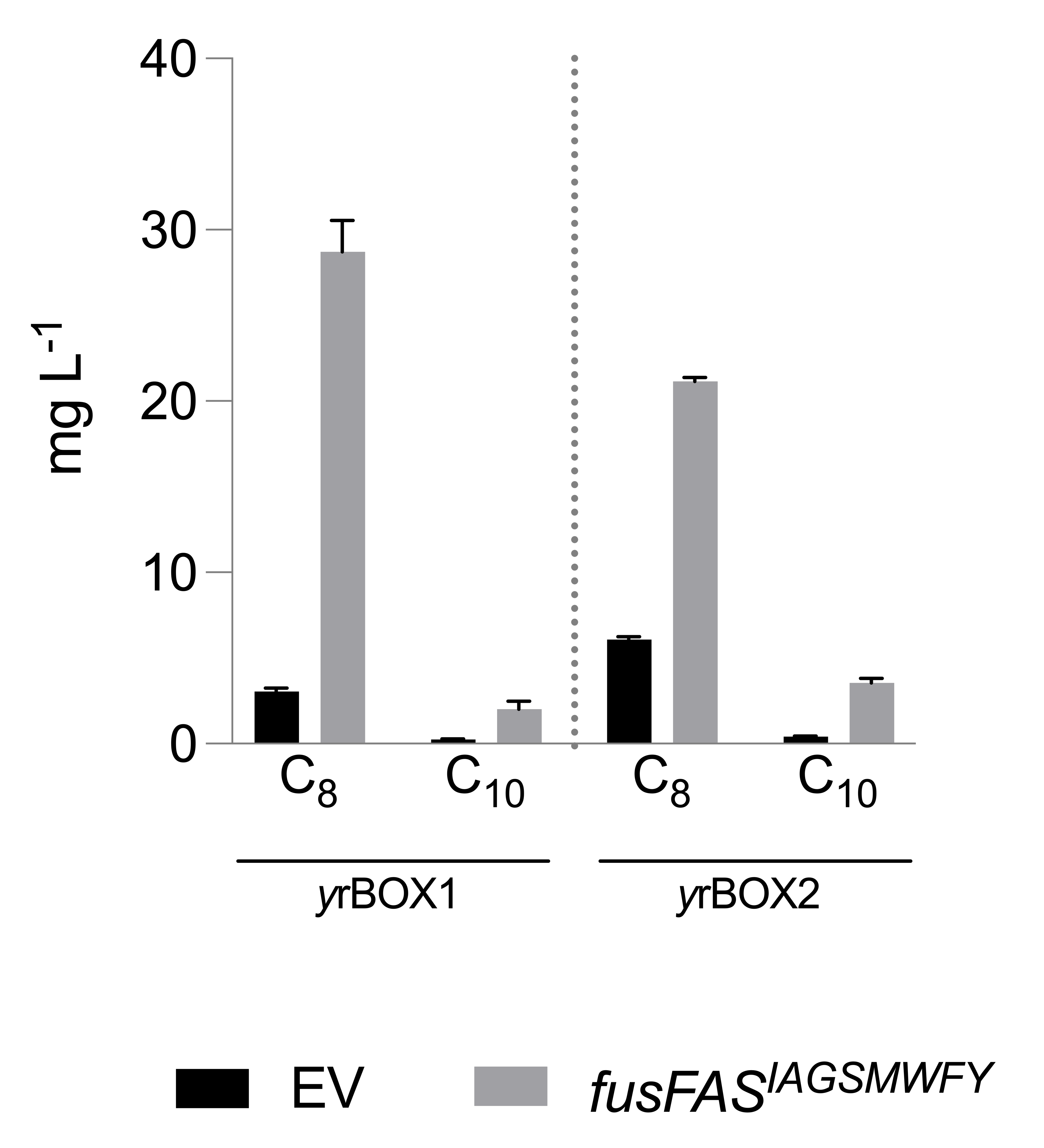

Supplement: Supplementary file 3 — Supplementary Figure 3. Plasmid-based overexpression of mutant fusFAS construct in 774 reverse β-oxidation strains [file 13068_2024_2586_MOESM3_ESM.tiff]
